# Radio Electric Asymmetric Conveyer (REAC) Neurobiological Stimulation Treatments in Dysfunctional Motor Behavior in Flail Arm Syndrome: A Case Report

**DOI:** 10.7759/cureus.28159

**Published:** 2022-08-19

**Authors:** Salvatore Rinaldi, Chiara Rinaldi, Arianna Rinaldi, Vania Fontani

**Affiliations:** 1 Research Department, Rinaldi Fontani Foundation, Florence, ITA; 2 Regenerative Medicine Department, Rinaldi Fontani Institute, Florence, ITA; 3 Neuroscience, Psychology, Drug Area, and Child Health (NEUROFARBA), University of Florence, Florence, ITA; 4 Adaptive Neuro Psycho Physio Pathology and Neuro Psycho Physical Optimization, Rinaldi Fontani Institute, Florence, ITA; 5 Biomedical Sciences, University of Sassari, Sassari, ITA

**Keywords:** reac, neuromodulation, neurostimulation, neurobiological stimulation, neurodegenerative diseases, amyotrophic lateral sclerosis, flail arm syndrome

## Abstract

Flail arm syndrome (FAS) is a variant of amyotrophic lateral sclerosis (ALS) that manifests itself with the progressive loss of motor control of the upper limbs starting from the proximal part. Both electrophysiological and magnetic resonance studies have shown that functional alterations in the subcortical structures, cerebellum, and cortex are present in this pathology. These alterations appear to play a significant component in determining cognitive, motor, and behavioral effects.

To try to modulate these alterations, in this case report, we used three noninvasive and specific neuromodulation treatments of the Radio Electric Asymmetric Conveyer (REAC) technology. The Neuro Postural Optimization (NPO), the Neuro Psycho Physical Optimization (NPPO), and the Neuro Psycho Physical Optimization Cervico-Brachial (NPPO-CB) with the aim of improving motor control, depression, anxiety, and stress. At the end of the treatment cycle that lasted five consecutive days, the patient regained the ability to raise his arms, a capacity he had lost for several months. This case demonstrates that REAC neurobiological modulation treatments aimed at improving dysfunctional neuropsychomotor behavior (DNPMB) can be useful in highlighting and reducing these components, allowing for better evaluation of the real neurodegenerative damage and determination of a better quality of life for these patients.

## Introduction

Flail arm syndrome (FAS) is a rare neurodegenerative disease [1]. FAS is a variant of Amyotrophic Lateral Sclerosis (ALS) [2,3] a disease involving progressive upper and lower motor neuron deterioration at multiple levels of the neuraxis [4]. The FAS was first described by Vulpian in 1886 as a syndrome of proximal weakness and hypotrophy of the upper limbs. FAS over the years has been identified with various names such as Vulpian-Bernard syndrome [5], Hanging arm syndrome, Man in the barrel syndrome, and Brachial amyotrophic diplegia.

FAS typically manifests with progressive weakness in the upper limbs and hypotrophy which is often symmetrical and proximal, with the appearance of signs of first motor neuron involvement, but without the occurrence of hypertonia or clonus.

Electrophysiological studies indicate that cortical and peripheral hyperexcitability is present, as well as in the typical form of ALS [6]. Furthermore, the presence of functional alterations in the subcortical structures, cerebellum and cortex has been demonstrated in these patients. These alterations appear to be responsible for the cognitive and behavioral symptoms associated with ALS [7-9]. Unfortunately, to date, it is not known whether these dysfunctions have occurred after the manifestation of the disease or may be one of the causes that trigger it.

In the case described in this study, to try to modulate the dysfunctional neuro psycho motor behavior (DNPMB) that can aggravate the clinical picture of the FAS, we used three noninvasive and specific neuromodulation treatments of the Radio Electric Asymmetric Conveyer (REAC) technology. The first is called Neuro Postural Optimization (NPO) with the aim of functionally reorganizing the functional cerebrum - cerebellum activity and improving motor control [10-12]. The second is called Neuro Psycho Physical Optimization (NPPO), and the third is called Neuro Psycho Physical Optimization Cervico-Brachial (NPPO-CB) with the aim of improving the state of depression, anxiety, and stress [13,14], which can negatively affect the clinical picture.

## Case presentation

Male patient 70 years old, in therapy with Riluzole. The patient has always practiced various sports activities, even in an intense way. Now he continues to play sports in which only the legs are involved. The first symptoms of FAS were manifested by a feeling of fatigue in the shoulders, which initially did not compromise the movements. In a few months, the symptoms progressively worsened causing a serious functional limitation in both arms but significantly more in the right arm. At the time of our first observation, functional limitation at the proximal level of the right upper limb did not allow the arm to be raised and compromised accustomed movements such as drinking or bringing food to the mouth. The patient was also examined for the presence of functional dysmetria [10,11,15] (FD), which manifests itself as an asymmetrical activation of symmetrical muscle groups, in our case at the level of the quadriceps femoris. FD can be related to acquired disorganization of the neuro-psycho-motor control in relation to the allostatic load [16,17]. The observed FD was approximately 8 centimeters, far exceeding the mean values observed in the general population. To correct the FD the patient underwent REAC NPO treatment. Immediately after the treatment, probably in relation to a reduction in cortical hyperactivity, the patient reported a different perception of the shoulders and in particular the subjective feeling of being able to better control the movements, even if he was unable to raise his arms. After the preliminary REAC NPO treatment, the patient underwent a cycle of treatments with NPPO and NPPO-CB, administered in consecutive succession in four distinct sessions, each separated by at least an hour's interval, for a total of 18 sessions in consecutive five days.

At the final check-up at the end of the eighteen sessions, the patient showed an important functional recovery in being able to raise both arms, in particular the left, which represented the most suffering (Video 1 and Video 2).

**Video 1:** The video, recorded immediately at the end of the REAC treatment cycle, shows the initial recovery of the patient's ability to raise both arms in succession.

**Video 2:** The video, recorded immediately at the end of the REAC treatment cycle, shows the initial recovery of the patient's ability to raise both arms at the same time.

## Discussion

FAS is a form of ALS, more common in males and with an apparently longer survival prognosis than in other ALS patients [1]. Although FAS is already in itself a serious neurodegenerative disease that compromises movements, this does not exclude that the motor clinical picture [7] can be conditioned and aggravated by DNPMB [7-9]. Studies have shown that the dysfunctionality of subcortical structures, cerebellum, and cortex play a role in determining the clinical picture. Furthermore, it seems that these dysfunctions contribute to determining a modification of the motor behavioral aspects and also in the ideation of the movement. To date, it is not known whether these dysfunctions are previous, concomitant, or subsequent to the onset of the disease.

The DNPMB in an unconscious way constantly conditions any motor activity of our body [18]. Being unconscious the DNPMB is difficult to identify and recognize both by the subject and by clinicians. Therefore, the clinical evaluation of the presence of DNPMB can represent an element of strategic importance in the clinical evaluation of subjects with neuromotor and neurodegenerative pathologies.

The case presented in this report is an example, as the neurobiological modulation treatments REAC NPO, NPPO, and NPPO-CB, were aimed exclusively at modulating the dysfunctionality of subcortical structures, cerebellum, and cortex can improve motor control and the depressive, anxious and stress state.

Although the treatments used in this case are unlikely to have any interference with the course of the disease, the strength of this study lies in two aspects. The first is further demonstration that NPO, NPPO, and NPPO-CB neuromodulation treatments are useful in treating dysfunctional adaptive motor and behavioral disorders. The second is constituted by the fact that these REAC treatments can highlight the DNPMB component, allowing to distinguish the dysfunctional component from the lesional component, which together manifest themselves in the clinical picture. The limitations of these observations are the fact of not knowing how long these effects will last over time without further cycles of REAC treatments and how much the inevitable worsening of the pathology will reduce the effects obtained.

## Conclusions

This case report suggests paying particular attention to the search for dysfunctional adaptive components, which by conditioning the clinical picture can significantly influence the diagnosis and prognosis of any pathological picture. Furthermore, this case report highlights how the REAC treatments used can help clinicians analyze how much the dysfunctional adaptive components are affecting the clinical picture.
